# Believer-Skeptic Meets Actor-Critic: Rethinking the Role of Basal Ganglia Pathways during Decision-Making and Reinforcement Learning

**DOI:** 10.3389/fnins.2016.00106

**Published:** 2016-03-24

**Authors:** Kyle Dunovan, Timothy Verstynen

**Affiliations:** ^1^Department of Psychology, University of PittsburghPittsburgh, PA, USA; ^2^Center for the Neural Basis of Cognition, University of Pittsburgh and Carnegie Mellon UniversityPittsburgh, PA, USA; ^3^Department of Psychology, Carnegie Mellon UniversityPittsburgh, PA, USA

**Keywords:** basal ganglia, reinforcement learning, decision making, diffusion model, exploration-exploitation tradeoff, speed-accuracy tradeoff

## Abstract

The flexibility of behavioral control is a testament to the brain's capacity for dynamically resolving uncertainty during goal-directed actions. This ability to select actions and learn from immediate feedback is driven by the dynamics of basal ganglia (BG) pathways. A growing body of empirical evidence conflicts with the traditional view that these pathways act as independent levers for facilitating (i.e., direct pathway) or suppressing (i.e., indirect pathway) motor output, suggesting instead that they engage in a dynamic competition during action decisions that computationally captures action uncertainty. Here we discuss the utility of encoding action uncertainty as a dynamic competition between opposing control pathways and provide evidence that this simple mechanism may have powerful implications for bridging neurocomputational theories of decision making and reinforcement learning.

## Introduction

Consider the scenario of being presented with a plate of cookies. You first grapple with the decision as to whether or not you even want a cookie, depending on your fortitude at maintaining dietary goals. After a brief deliberation you decide to make an exception to your diet and start to reach toward the plate, however during the reach you realize that what you thought was a chocolate chip is in fact a spider resting on top, prompting you to reactively cancel your movement. The experience of seeing the spider also impacts the certainty that you will reach for a cookie in the near future, making you more cautious and increasing your chances of sticking to your diet. This adaptability of both proactive (i.e., breaking your diet) and reactive (i.e., responding to the spider) behavioral control, in the face of multiple sources of uncertainty, is one of the most evolutionarily important functions of the mammalian brain.

**Figure 1:**
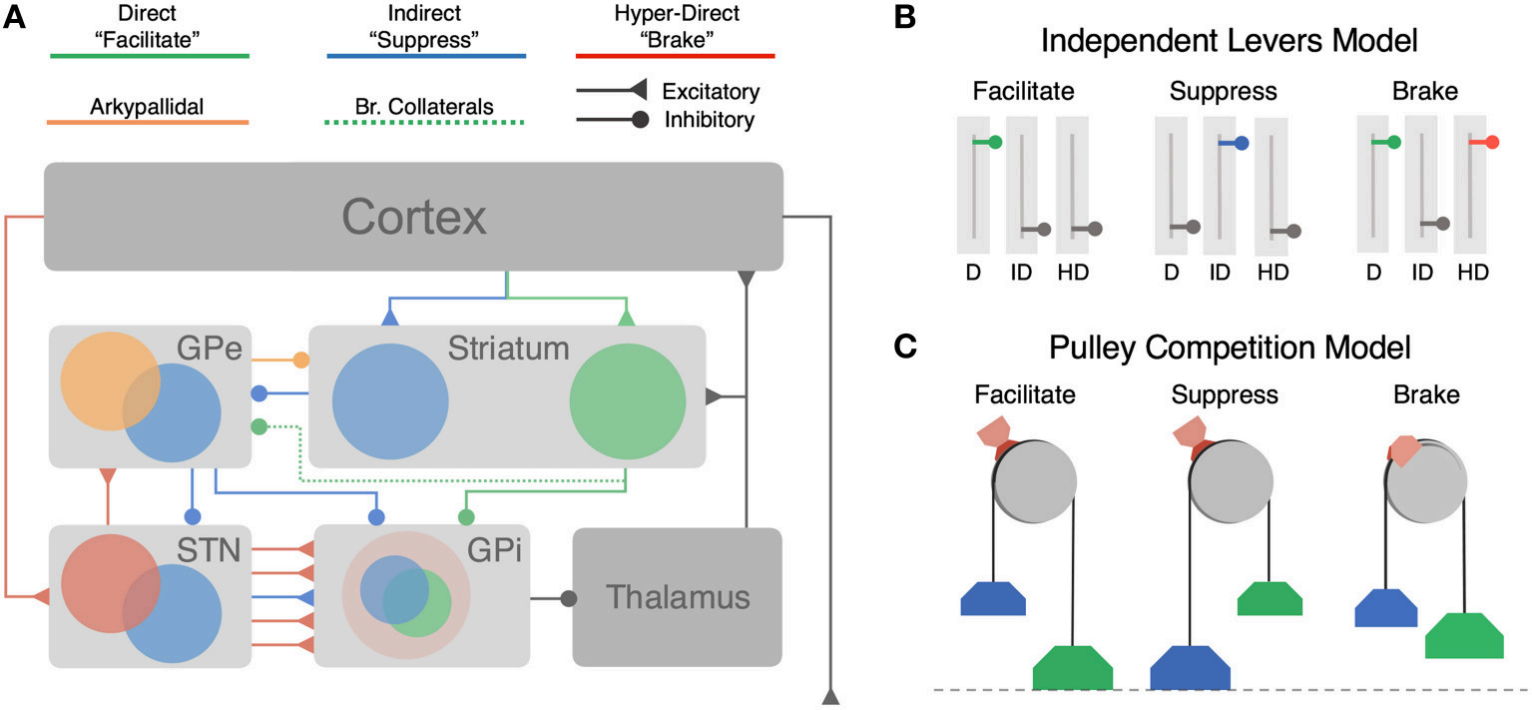
**Architecture of cortico-BG pathways and hypothesized functional models. (A)** Cortico-BG pathways including three major inputs to the striatal direct (green), indirect (blue) pathways, and the subthalamic hyper-direct (red) pathway. Bridging collaterals (green, dotted) connect the direct pathway to the indirect pathway via projections to the GPe. The arkypallidal pathway (orange) sends inhibitory feedback projections from the GPe to the striatum. Both the direct pathway (cortex-striatum-GPi) and “short” indirect pathway (cortex-striatum-GPe-GPi) form focused projections throughout the network corresponding to individual action channels. The “long” indirect pathway (cortex-striatum-GPe-STN-GPi) and hyper-direct pathway (cortex-STN-GPi) deliver diffuse excitatory inputs to the output nucleus. **(B)** Independent Levers Model (i.e., the canonical model) assumes that the direct (left, green), indirect (middle, blue), and hyper-direct (right, red) pathways are structurally and functionally segregated. Each pathway is operated in isolation for facilitating, suppressing, or braking motor output in the BG. **(C)** Pulley Competition Model (i.e., Believer-Skeptic) assumes that the direct and indirect pathways compete throughout the BG (see Section Introduction), with the strength of each pathway acting as weights on opposing sides of a pulley. As activation in the direct pathway overpowers that of the indirect pathway, this imbalance accelerates the network toward “facilitation,” resulting in an executed action when the difference reaches a critical threshold (dotted line). In the event of a stop cue, the action can be reactively canceled if the pulley brake (red brake pad) is activated before the direct-indirect difference reaches a critical threshold. The accelerating (e.g., nonlinear) dynamics of an imbalanced pulley lead to less efficacious braking when the network is pulled further toward action execution (e.g., longer brake streaks on pulley wheel). This dependency illustrates how proactive modulation of the direct-indirect balance may influence reactive stopping via activation of the hyper-direct pathway.

Several lines of evidence point to a central role of cortical and basal ganglia (BG) circuits in modifying action decisions in dynamic environments; however, the mechanisms by which cortico-BG pathways encode uncertainty and adapt with experience remains controversial. This controversy is fueled by a history of often inconsistent and sometimes paradoxical experimental findings. Central to this debate is the canonical model of the BG (Albin et al., 1989; DeLong, 1990), where action selection is determined by the dynamics of three separate control pathways (Figure 1A): the direct pathway (Figure 1A; green) that facilitates motor output, the indirect pathway (Figure 1A; blue) that suppresses motor output, and the hyper-direct pathway (Figure 1A; red) that mediates fast cancelation of sub-threshold motor decisions. According to the canonical model, all three pathways act as independent decision processes that regulate subsequent thalamic output to cortex (DeLong, 1990).

The architecture of the BG is such that each control pathway converges on a common output nucleus, suggesting that at some level these pathways may interact. Indeed recent electrophysiological (Mallet et al., 2012; Cui et al., 2013; Kress et al., 2013; Cazorla et al., 2014), neuroimaging (Chikazoe et al., 2009; Jahfari et al., 2011, 2012), computational (Bahuguna et al., 2015; Dunovan et al., 2015; Gurney et al., 2015; Wei et al., 2015), and behavioral (Verbruggen et al., 2014) findings have cast doubt on the traditional independent process framework, in favor of a dependent process model where all three pathways compete for control over motor output. These observations allude to a novel reconceptualization of the BG where the competitive dynamics between all three pathways reflect a weighted combination of learning and decision variables (Cazorla et al., 2014; Bahuguna et al., 2015; Dunovan et al., 2015; Gurney et al., 2015; Wei et al., 2015). This provides a theoretically valuable premise for characterizing BG involvement in adapting actions in uncertain environments.

Here we explore the computational utility of a dependent process model of BG pathways. This review is partitioned into three sections. First, we provide an in depth summary of current debates regarding the role of the BG in inhibitory control. Next, we discuss recent advances relating computational models of decision-making and reinforcement learning to activity in cortico-BG networks. Finally, we propose a framework for synthesizing control, decision making, and learning within BG circuits, arguing that these pathways are best characterized by their ability to integrate uncertainty into goal-directed actions.

### Interactions between direct and indirect pathways

According to the canonical BG model, in the cookie scenario described above the decision to reach for the cookie is driven by cortical activation of the direct pathway, whereas the decision to abstain is driven by activation of the indirect pathway. These two control signals are traditionally thought to occur in isolation of one another, such that upstream cortical regions either facilitate actions by activating the direct pathway or suppress actions by activating the indirect pathway (Hikida et al., 2010). More recently, the canonical model has been revised to include a third “hyper-direct” pathway in which cortical excitation of the subthalamic nucleus (STN) applies strong, diffuse suppression of action-facilitating signals in the direct pathway when a cue to stop (e.g., spider) is detected in the environment. This pathway is thought to race against action facilitating signals in the direct pathway in order to cancel an inappropriate or unnecessary action (Aron and Poldrack, 2006). Together the dynamics of the direct, indirect, and hyper-direct pathways form the basic building blocks of behavioral control through BG pathways.

The canonical BG model fundamentally assumes that all three control pathways run in parallel to each other and do not interact. Thus, the direct and indirect pathways may be viewed as two independent levers that are recruited in order to select appropriate actions that are in line with current behavioral goals (Figure 1B). This is often referred to as “proactive” control (Braver, 2012). The hyper-direct pathway also acts as an independent lever, but one that is recruited “reactively” upon detection of an environmental stop cue rather than endogenous goals (Aron and Poldrack, 2006). That is, the hyper-direct pathway acts as a safety brake for situations that require late action cancelation, whereas the indirect pathway serves to selectively suppress actions that conflict with the current goals.

The notion that cortico-BG pathways operate as independent control mechanisms during action selection is reinforced by a large body of evidence demonstrating their opposing effects on motor output (see Albin et al., 1989 for review of basal ganglia motor circuitry and Calabresi et al., 2014 for an updated view). Recently, Kravitz et al. (2010) showed that optogenetic stimulation of direct pathway medium spiny neurons (dMSN) facilitated locomotor behavior in mice, whereas stimulation of indirect pathway MSN's (iMSN) led to motoric freezing. This was interpreted as strong evidence for the existence of structurally and functionally separate pathways for facilitating and suppressing movement. In contrast with the findings of Kravitz et al. (2010) a recent study by Cui et al. (2013) showed that both direct and indirect MSNs in the mouse dorsal striatum increase their firing just before contraversive movements. These findings provide the first clear evidence of a long theorized (Alexander and Crutcher, 1990; Mink, 1996), but empirically unfounded, action selection mechanism in the BG whereby cortical projections activate the direct pathway of a target action while simultaneously activating the indirect pathway of competing actions. Intuitively, this form of “center-surround” selection (Mink, 1996) becomes increasingly advantageous when there are many alternative actions from which to choose, acting as a safeguard against co-expression of multiple, interfering outputs. In this context, the observation that direct and indirect pathways are activated in unison marks an important discovery, but one that is still consistent with the independent levers model. Both pathways retain the same opposing influence over motor output and are operated independently and exclusively within each action channel. In contrast with this view, however, goal-directed learning coincides with bidirectional plasticity at cortico-striatal synapses, increasing the excitability of dMSNs while suppressing the excitability of iMSNs (Shan et al., 2014). This would suggest that, rather than behaving as independent levers (Figure 1B), the direct and indirect pathways act as weights on opposing sides of a pulley that bias the network toward a more facilitating or suppressing state for a given action (Figure 1C). Over the course of learning, more weight is added to the direct pathway of sensorimotor mappings that yield positive results whereas weight is added to the indirect pathway of aversive mappings (discussed in detail in Section Dopaminergic Modulation of Believer-Skeptic Balance). This competitive balance also interacts with the efficacy of the hyper-direct pathway, acting as a safety brake on the pulley that, if applied soon enough, can prevent the weight of the direct pathway from overcoming the weight of the indirect pathway (Figure 1C, right).

Architecturally there is ample evidence to suggest that BG pathways interact with each other. Most notably, all three pathways converge at the output nucleus of the BG, the internal segment of the globus pallidus (GPi) in humans and substantia nigra pars reticulata (SNr) in rodents. This region is generally considered to represent the locus of determination for action decisions. At rest the GPi tonically inhibits the thalamus, marking an important property of BG circuitry in that the default state of the network is motor suppressing. Thus, in order to elicit a motor output, the direct pathway must sufficiently inhibit target cells in the GPi in order to disinhibit the corresponding channel in the thalamus, that “opens the gate” for the appropriate action output (Mink, 1996). In situations requiring the inhibition of an action, indirect and hyper-direct pathways prevent motor output by strengthening pallido-thalamic inhibition so as to override the action gating effects of the direct pathway. In the canonical indirect pathway model, cortical inputs to striatal iMSNs inhibit tonic firing of neurons in the external segment of the globus pallidus (GPe), thereby suppressing motor output by further disinhibiting outputs in the GPi (Figure 1A, short indirect pathway) and enhancing excitatory output of the STN (Figure 1A, long indirect pathway). Given that all three of the major BG pathways show signs of convergence in the GPi (Smith et al., 1998; Mathai and Smith, 2011), it is easy to see how they could compete for a final decision output from the BG to the motor thalamus. It is also worth noting that cortical inputs to these pathways are not as segregated as previously thought. For instance, both dMSNs and iMSNs receive convergent thalamic (Huerta-Ocampo et al., 2013) and cortical inputs (Kress et al., 2013; Wall et al., 2013; Haber, 2014). Although there is a reliable tendency for prefrontal and frontal motor cortices to innervate iMSNs and for sensory and limbic cortices to innervate dMSNs (Wall et al., 2013), suggesting that there is some degree of segregation of information depending on the source of the cortical inputs.

In addition to the convergence of pathway inputs and outputs, a growing body of evidence has emerged revealing pathway-level interactions in the feedback loops mediated by distinct sub-populations in the GPe. For instance, the GPe of the indirect pathway has been shown to send feedback projections to the striatum (Mallet et al., 2012) that synapse onto both major MSN subtypes, as well as striatal fast-spiking interneurons, or FSIs (forming up to 13,000 synapses each; Silberberg and Bolam, 2015). A recent study (Mallet et al., 2016) found that this feedback pathway, termed the arkypallidal pathway (Figure 1A; orange), was engaged on successful “stop” trials in a reactive control task. The authors concluded that the arkypallidal pathway is responsible for silencing descending motor commands in the striatum, acting in parallel with hyper-direct “braking” of GPi output to cancel a planned response. In stark contrast with this conclusion, another study found that arkypallidal neurons displayed the strongest activation during the execution, not cancelation, of an action (Dodson et al., 2015). This is consistent with computational studies proposing that arkypallidal feedback could facilitate motor output by suppressing FSIs (Bahuguna et al., 2015) since FSIs preferentially target dMSNs over iMSNs (Mastro et al., 2014).

Given that arkypallidal projections are known to innervate both motor suppressing and facilitating populations (Silberberg and Bolam, 2015), these seemingly discordant findings are suggestive of a more modulatory role in *action selection* rather than execution or cancelation, *per se*. In line with this assessment, single unit recordings in the macaque GPe have revealed two functionally distinct sub-populations that contribute to anti-saccades in the countermanding task (Yoshida and Tanaka, 2016): one that decreases firing, consistent with the response of prototypical indirect pathway neurons during selective action suppression, and another that increases firing, rising maximally before successful anti-saccades. One intriguing possibility is this activity-*increasing* population represents activation of the arkypallidal pathway by the excitatory inputs from the STN. The resulting feedback into the striatum could facilitate rapid activation of a previously unplanned response by suppressing FSIs which preferentially silence dMSNs. Thus, despite having a generally dampening effect on both MSN subtypes, arkpallidal feedback would give dMSNs an advantage for responding to strong channel specific input from cortex, unimpeded by activation of that channel's indirect pathway. This modulatory mechanism is more parsimonious than the channel specific activation and suppression mechanisms proposed by previous studies and is consistent with the known diversity of striatal cell types targeted by this pathway (Silberberg and Bolam, 2015).

Finally, there is one architectural feature that has explicitly been shown to mediate an interaction between direct and indirect pathways: a significant portion of dMSNs send bridging collaterals to the GPe, acting as indirect pathway efferents (Figure 1A, dotted green line; Wu et al., 2000). A recent study by Cazorla et al. (2014) found that dMSN bridging collaterals are proliferated by promoting indirect pathway activity via D2R-upregulation in iMSN's. The authors demonstrate through a series of experiments how experience-dependent changes in bridging collateral density alter the physiological and behavioral dynamics associated with direct and indirect pathway activation. In stark contrast with independent levers model, Cazorla et al. (2014) found that optogenetic stimulation of the direct pathway coincided with a moderate number of inhibited cells in the GPe in control mice, demonstrating clear interaction between direct and indirect pathways in normally developed animals. Remarkably, this effect became more salient with the activity-dependent proliferation of collaterals into the indirect pathway and actually reversed the effect of the direct pathway activation on behavior—suppressing locomotion rather than facilitating it.

One major implication of the Cazorla et al. (2014) study is that frequently suppressed actions, such as those that are costly or uncertain, become more difficult to execute as cortical activation of the direct pathway is restricted by proliferated dMSN collaterals into the indirect pathway. This functional link between direct and indirect pathways could potentially explain numerous conflicting findings in electrophysiological and human neuroimaging studies. For instance, both pathways, when stimulated in isolation, lead to heterogeneous (increased and decreased) changes in the firing of downstream GPi/SNr cells (Freeze et al., 2013), whereas others (Kravitz et al., 2010) have demonstrated clearly opposing behavioral effects following direct (e.g., facilitation) and indirect (e.g., suppression) pathway stimulation. These seemingly inconsistent findings can be reconciled by revising the canonical model to incorporate cross-talk between the direct and indirect pathways, either through direct-pathway bridging collaterals or through arkypallidal feedback projections to the striatum. Finally, human neuroimaging studies of response inhibition have proposed that proactive control is singularly driven by cortical activation of striatal indirect pathway (Majid et al., 2013). The findings by Cazorla et al. (2014), in addition to many of the findings discussed above, strongly caution against the notion that proactive control arises from exclusive engagement of the indirect pathway or that modulation of this control is limited to cortical sources.

## Believer-skeptic: Encoding uncertainty as a dynamic competition

The studies discussed thus far provide evidence against the independent lever model of cortico-BG pathways and instead favor a model in which these pathways engage in a dynamic competition: as activity increases in one of the pathways the balance is upset and the network accelerates toward motor-facilitating or motor-suppressing state. Seen in this light, this direct-indirect competition represents a potentially important decision-making mechanism whereby multiple sources of uncertainty can be weighed and integrated before choosing between potential actions. In this way, the direct–indirect competition implements a decision by weighing the arguments of a Believer (e.g., direct pathway) against those of a Skeptic (e.g., indirect pathway). Because the default state of the BG is heavily motor suppressing (Bahuguna et al., 2015), the burden of proof falls on the Believer and thus actions are only executed when the accrued evidence sufficiently reduces the Skeptic's uncertainty. Here, we show that the competition between the direct and indirect pathways can be formalized by the dynamics of a simplified neural network model of cortico-BG pathways and mapped onto parameters of accumulator models of decision-making. From this, we argue that the competitive nature of cortico-BG pathways is a critical feature for encoding uncertainty and adapting behavior in changing environments.

### Competing BG pathways encode decision uncertainty

Computational models of decision-making predominantly fall within the broader class of accumulation-to-bound models, in which a decision is computed by accumulating the evidence for one choice over another until a threshold is met and a choice can be made. When deciding between two alternative hypotheses, or choices, the optimal rate of accumulation and other decision criterion for maximizing speed and accuracy is described by the Drift-Diffusion Model (DDM; Ratcliff, 1978; see Ratcliff and McKoon, 2008 for a review). Successful application of this model to a broad spectrum of behavioral phenomena has established the DDM as the archetypal model of decision-making. By fitting models to behavioral data, response-time and accuracy measures are decomposed into hypothesized subcomponents of their generative mechanism that are quantified by specific model parameters. These parameters can then be used to extract or predict neural activity related to individual subcomponents of the decision process. While significant progress has been made by leveraging stochastic accumulator models to aid in the prediction and interpretation of data in experimental neuroscience, it remains an open question at what level of neural processing (i.e., single neurons, local circuits, networks) these parameters are realized in the brain.

Studies investigating the neural basis of decision-making have largely focused on frontal and parietal systems, following from early observations that single-neurons in these regions appear to display the same ramp-to-threshold characteristics as the DDM. More recently, it has become clear that the neural processes involved in decision-making are much more distributed than previously thought, suggesting that decision variables are tracked by populations of neurons (Park et al., 2014) at both the cortical (Heitz and Schall, 2012, 2013) and subcortical (Ding and Gold, 2012b, 2013) levels. Indeed, mounting evidence points to the BG as a critical part of the decision network, serving as a convergence zone for contextual and sensory information prior to decision commitment (Ding and Gold, 2012b; Nagano-Saito et al., 2012; Yanike and Ferrera, 2014; Dunovan et al., 2015; Keuken et al., 2015; Wei et al., 2015). Most of the cortical regions that have been implicated in the evidence accumulation process send direct projections into the BG (Haber et al., 1995; Draganski et al., 2008; Averbeck et al., 2014; Verstynen, 2014; Jarbo and Verstynen, 2015), as do many other context- and performance-monitoring regions (Haber et al., 1995; Forstmann et al., 2012; King et al., 2012; Haynes and Haber, 2013). This convergence of cortically distributed decision signals into the BG adds credence to the growing body of evidence suggesting this network is critical for imposing a threshold on accumulating decision evidence (Lo and Wang, 2006; Forstmann et al., 2008; Bogacz et al., 2010; Cavanagh et al., 2011; Mansfield et al., 2011; Bahuguna et al., 2015; Frank et al., 2015; Wei et al., 2015).

It is important to note that, in contrast with the DDM-like ramping of cortical accumulators, the neural implementation of a decision threshold is unlikely to present in such a straightforward manner (Simen, 2012; Heitz and Schall, 2013). Changes in the decision threshold of the DDM can capture decision-related computations that occur at various stages of processing; for instance, a decrease in the DDM threshold can describe the behavioral effects of indiscriminately increasing the baseline of evidence for both alternatives prior to sensory input. Furthermore, a shift in the baseline of evidence may reflect priming in either low-level sensory regions, downstream evidence accumulators, cortical and subcortical motor circuits, or some combination of all of these domains. Indeed, Heitz and Schall (2012, 2013) have shown in a series of computational and electrophysiological studies that, behavioral adjustments optimally explained by a change in decision threshold in standard accumulator models arise from a combination of parameter changes in the of neurons in the frontal eye fields (FEF). According to these findings, the representation of the decision threshold in standard accumulator models is best thought of as an abstraction of more sophisticated network dynamics underlying speed-accuracy tradeoffs. Therefore, it is useful for the purposes of this review to clarify the meaning of *decision threshold* in the abstract sense so as to distinguish this meaning from the mechanism by which it is theoretically modeled or neurally implemented. At a conceptual level, a decision threshold can be thought of as the “switch” or “latch” mechanism responsible for transitioning from an accumulation state to an action execution state. In contrast to the notion of a threshold as the “upper limit” or “criterion boundary” placed on evidence accumulation, switches are dynamic processes themselves and can be adjusted to be more or less sensitive to perturbation.

Converging electrophysiological (Schall et al., 2011; Ding and Gold, 2012a) and computational (Simen, 2012; Standage et al., 2014) evidence suggests that competing populations of neurons can implement a transition threshold in the presence of sufficient nonlinearity in the competitive inhibition between populations. For instance, Schall et al. (2011) proposed the gated-accumulator model to account for the cross-inhibition between target and distractor populations in the FEF. These authors trained macaques to maximize reward by emphasizing the speed or accuracy of their performance in a visual search task based on a prior cue. Behavioral speed-accuracy tradeoffs were well described by a traditional accumulator model allowing only the threshold to vary across conditions. However, recordings in choice-selective FEF neurons displayed simultaneous changes in the baseline, onset, and rate of firing as a function of decision policy. Consistent with this, Wei et al. (2015) recently showed that competitive dynamics between the direct and indirect pathways in a spiking neural network of the BG could be tuned to strategically adjust the decision threshold. In their model, changing the synaptic efficacy of indirect pathway output from the striatum to the GPe effectively modulated the threshold at which accumulating cortico-striatal inputs produced an action. Thus, rather than manifesting as a change in the RT-locked firing rate of cortical accumulators (as might be expected if neural decision thresholds were implemented as in the DDM), this model showed that BG circuitry can approximate the same mechanism by modulating balance of the direct and indirect pathways.

**Figure 2:**
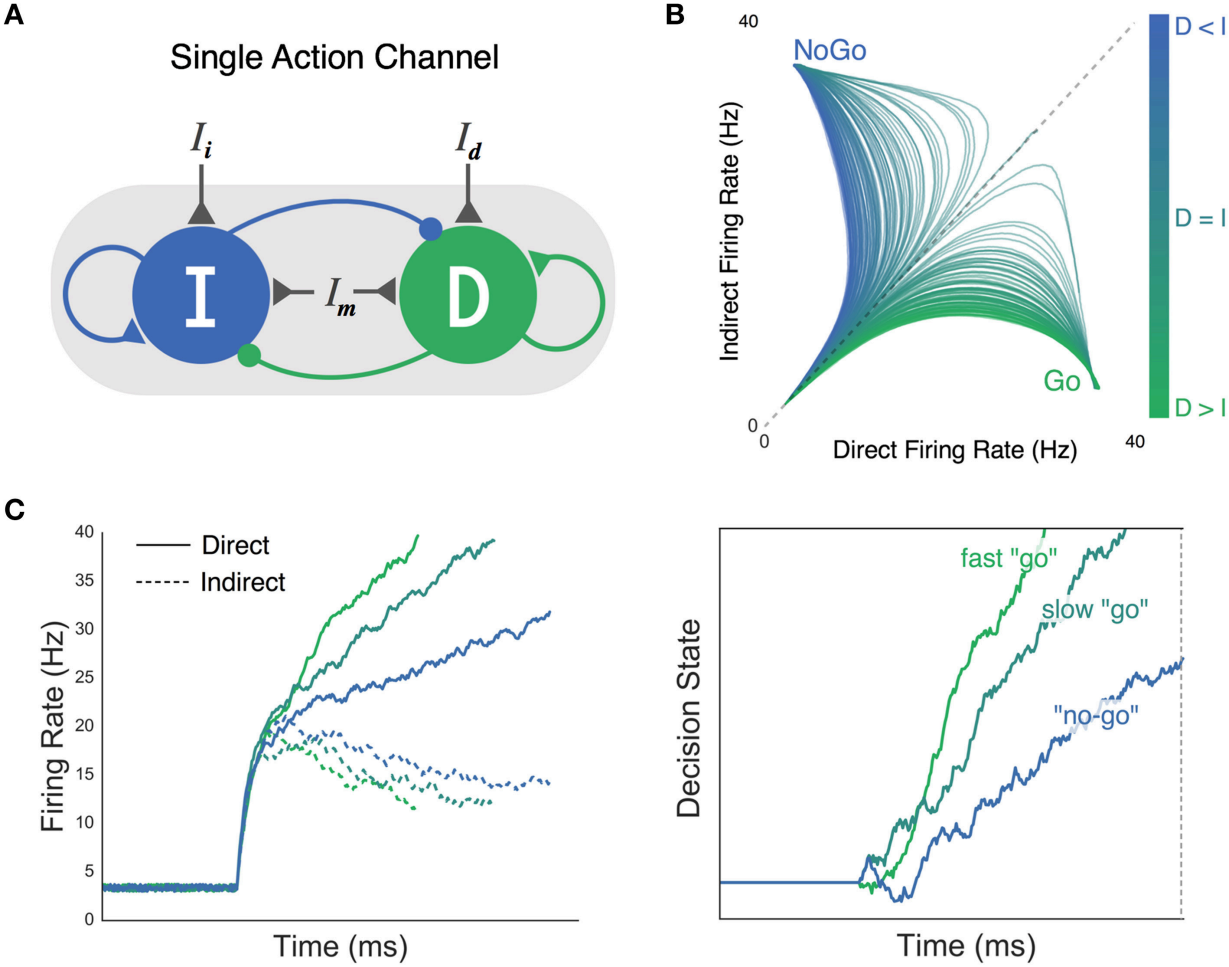
**Believer-Skeptic framework and competition between the direct and indirect pathways in neural and decision space. (A)** The direct (D) and indirect (I) pathways are modeled as two competing (i.e., mutual inhibition) accumulators with recurrent self-excitation reflecting population attractor dynamics. Selective input to the direct (I_d_) and indirect (I_i_) pathways is weighted and summed with input from a modulatory (non-selective) population (I_m_) which controls the baseline excitability of the network. **(B)** Network state plotted as a function of different ratios of direct and indirect pathway activation. Greater activation of the indirect pathway leads to fast attraction toward a NoGo state (more blue, motor suppressing), whereas greater activation of the direct pathway attracts the network toward a Go state (more green, motor facilitating). **(C) Left panel**: firing rates of direct (solid lines) and indirect pathways (dotted lines) plotted across time for different ratios of input (I_d_:I_i_). **Right panel**: accumulation of decision evidence toward an execution threshold, reflecting the normalized difference of the direct and indirect pathways in the left panel. High I_d_:I_i_ ratio accelerates the rate of evidence accumulation, leading to a fast “go” decision (green). As this ratio is reduced (bluish-green), weaker attraction by the direct pathway manifests as a slower rate of accumulation, producing a “no-go” decision when evidence fails to reach threshold by a deadline (blue).

Similar in concept to the gated accumulator model, consider the simple neural network shown in Figure 2A, that is composed of two competing neural populations with recurrent excitatory connections. The mutual inhibitory connections between populations of direct and indirect units, in combination with recurrent self-excitation, leads to a non-linear change in the separation of their firing rates over time. The point in time at which this separation occurs marks the “gate” in the gated accumulator model. Network properties that promote early gating correspond to a lower threshold in the traditional DDM. That is, they both reduce allotted time for evidence to be gathered. On the other hand, the effective threshold can be “raised” by increasing the time constant of evidence accumulation, reflected in the network as a delayed gate or more gradual separation of competing population activity.

While the gated accumulator model was originally used to capture activity in target and distractor populations of cortical neurons (Schall et al., 2011), we propose that a similar threshold mechanism is implemented by a competition between direct and indirect pathways in the BG. In this reduced form of the model, the respective strength of each pathway is determined by several factors, including the amount of cortical input to each population, the weight applied to those inputs (i.e., cortico-striatal synaptic efficacy), and the overall excitability of the network based on non-specific modulatory inputs. Thus, rather than the two populations in Figure 2A representing target and distractor stimuli, they represent a single action channel composed of a Believer population that competes with a Skeptic population for control over motor output. It is important to point out that, in contrast with the specific subpopulations of FEF neurons depicted in the gated accumulator, this general attractor network is not meant to depict specific populations of cells (i.e., dMSNs, iMSNs, etc.) or specific projections between or within BG nuclei (i.e., arkypallidal pathway, branching collaterals, etc.,). Rather, we have opted to focus on the implications of within-channel competition between motor-facilitating and suppressing dynamics at the network level. Thus we are sacrificing certain physiological details for the sake of tractability in relating these dynamics to behavior. Co-activation of direct and indirect pathways within a given action channel has been proposed by previous models of BG pathways (Brown et al., 2004; Schroll and Hamker, 2013; Wiecki and Frank, 2013); however, strong empirical evidence for this competition is limited to recent electrophysiological studies. This may be due, in part, to the fact that dMSN and iMSN's are often not distinguished in single-unit studies of decision-related activity in striatum (Ding and Gold, 2010, 2012b, 2013). Still, recent confirmation that both pathways are active prior to movement (Cui et al., 2013) has largely been taken as evidence of a center-surround action selection mechanism (Friend and Kravitz, 2014) where the “go” lever of target channel is surrounded by the “no-go” levers of competing channels. Converging lines of evidence suggest that center-surround selection not only emerges from, but also requires simultaneous recruitment of direct and indirect pathways for each action. For instance, a recent computational study found that both pathways must be active to a controlled degree within all channels, otherwise no actions or too many actions are selected (Gurney et al., 2015). This contingency is in line with the bidirectional reweighting of cortical inputs to the direct and indirect pathways observed during the acquisition of goal-directed behavior (Shan et al., 2014). Accordingly, we propose that both pathways are activated for each individual action, but to varying degrees such that the ratio of direct-to-indirect activity is optimized during goal-directed learning. Under this assumption, a center-surround mechanism can still arise in which a target action enjoys a greater direct-to-indirect ratio than surrounding actions. In fact, there is good reason to think that actions are selected through a combination of center-surround suppression and the action-specific balance of facilitation and suppression. We elaborate more on this in the following section.

### Linking neural competition to accumulator models

The Believer-Skeptic framework presented here proposes that cortico-BG pathways implement a decision threshold as a dynamic competition of action facilitating and suppressing network states. While we propose this to be a more neurally plausible mechanism of threshold implementation than that presented in the DDM, this is not to say that model abstraction in the DDM is not useful. In fact, it is necessary for developing quantitative theories that can be meaningfully parameterized at cognitive and behavioral levels of description. In order for these models to be applied to neural data there must be an appreciation for the mapping between cognitive parameters and the more complex neural processes that they represent.

Within the standard DDM, “competition” is inherently captured by the accumulating decision process where each step up or down represents the instantaneous evaluation of two competing hypotheses: an action decision and its null alternative. In the context of basic perceptual decisions, stimuli with high signal-to-noise ratio (SNR) produce faster rates of evidence accumulation toward a decision boundary, and are thus recognized faster and more reliably than noisy stimuli. This is an important point to emphasize, as the unidirectional change in the speed and accuracy of decisions is what fundamentally distinguishes a change in drift-rate from a change in the decision threshold in the standard DDM. As hinted at earlier the decision process can instead be reparameterized to reflect different hypotheses regarding the neural processes responsible for integrating contextual information with sensory evidence (Standage et al., 2014). In the Believer-Skeptic framework, contextual information and sensory evidence converge as weighted cortico-striatal inputs to the direct and indirect pathways of a single action channel (Figure 2A). The strong recurrent dynamics within each pathway lead to bistability in the network output (Figure 2B), an important property for implementing a switch between two states. Even when the weighted input to each pathway is comparable, small amounts of noise can disrupt the balance enough to cause a state transition given sufficient self-excitation. As a result, both pathways initially increase their firing rate then diverge as activation in one pathway supersedes and inhibits the other, switching the network toward a “Go” or “NoGo” attractor state (Figure 2B). Thus, rather than the sensory driven drift-rate of the DDM, the moment-to-moment competition between alternative hypotheses in the Believer-Skeptic framework is driven by a weighted combination of contextual and sensory information. This form of competition can be seen in Figure 2C, in which Go-NoGo decisions are made by accumulating the output (right panel) of the direct-indirect competition (left panel) under different levels of contextual uncertainty. When action uncertainty is low, the network is accelerated toward a “Go” state (Figure 2B) by stronger activation of the direct pathway, causing a faster accumulation of decision evidence toward a fixed execution threshold. Neurophysiologically, the fixed upper threshold of decision evidence in Figure 2C (right plot) can be conceptualized as the level of pallidal suppression necessary to disinhibit the thalamus so that an action is executed.

We recently proposed a modified accumulator framework motivated by the general control dynamics of the Believer-Skeptic network in Figure 2, where action decisions are executed by accumulating evidence toward a fixed threshold in the presence of dynamic gain. In our so-called dependent process model, we found that contextual information (i.e., cued probability of reward) modulates the drift-rate of the execution process (as seen in the right panel of Figure 2C). As action uncertainty increases the drift-rate is suppressed, producing a “no-go” decision when this suppression prevents the decision process from reaching the execution threshold by the trial deadline (Dunovan et al., 2015). Based on the apparent structural overlap of BG pathways in the output nucleus (shown as overlapping red, blue, and green fields in the GPi of Figure 1A), we hypothesized that contextual modulation of competition between direct (i.e., Go) and indirect (i.e., NoGo) pathways should also influence the efficacy of the hyper-direct (i.e., Stop) pathway during reactive action cancelation (Jahfari et al., 2010, 2011, 2012), Indeed, behavioral fits to RT and choice data in a reactive stop-signal task favored a model in which contextual suppression of the execution drift-rate improves the efficacy of a nested but separate action cancelation process. Collectively, these findings show how the contextual uncertainty associated with a future action is not only critical for making a goal-directed decision about executing that action, but also complements the ability to reactively cancel it based on environmental feedback.

This dependent process model also captured physiological responses of BG pathways. By integrating the execution process across the trial window, we were able to capture the duration and magnitude of accumulating activity leading up to a decision. Integrating the execution process in this way effectively collapses the decision process into a single measure, similar to how the blood oxygen-level-dependent (BOLD) signal would filter the neural activity generated by attractor network in Figure 2A. Consistent with the behavioral fits, we found that contextual modulation of the drift-rate was able to capture the pattern of BOLD activity in the thalamus (the primary output target of the BG pathways) during “go” and “no-go” decisions across varying degrees of uncertainty. This finding is consistent with single-unit recordings of neurons in the macaque motor thalamus which show a similar RT-dependent ramp in firing rate prior to action execution (Tanaka, 2007; Masaki Tanaka and Kunimatsu, 2011).

One interpretation of this finding is that pre-action ramping in the thalamus is driven by the differential activation of upstream direct and indirect pathways and thus contextual modulation of this signal occurs by changing the weights of specific cortico-striatal connections or by altering background excitability in the striatum. The hypothesis that the striatum is where contextual information comes to bear on decision evidence is often contrasted with the hypothesis that this is accomplished by the thresholding function of the STN (Bogacz et al., 2010). That is, a change in the slope of thalamic firing rates could be due to decay in the hyper-direct activation of the STN, allowing pallidal suppression by the direct pathway to disinhibit the thalamus at a proportional rate. The distinction between striatal and STN control over decision threshold is a critical one (Bogacz et al., 2010), as these structures have very different input-output motifs that hint at disparate functional roles. The input-output organization of the striatum is thought to be channel-specific, propagating individual action-commands from cortex to corresponding units in the GPe (indirect) and GPi (direct) segments. The STN, on the other hand, receives converging afferents from cortex and the GPe and delivers diffuse excitatory drive to the GPi, suggesting this structure modulates the decision threshold in a non-specific manner for all actions under consideration.

In fact, another hypothesis has been proposed for the role of the STN in decision-making that both complements the role of the striatum in the Believer-Skeptic framework and distinguishes the functional relevance of indirect and hyper-direct activation of the STN. Bogacz and Gurney (2007) presented a neural network model in which the STN normalizes activity in the GPi to accommodate different set sizes of alternative choices. In their model, sensory evidence for each alternative is fed into a corresponding action channel in the striatum in parallel with projections that activate the STN. As a result, the cortico-striatal activation within each individual channel of the GPi (i.e., representing candidate actions “A”, “B,” and “C,” for instance) is represented as a proportion of the evidence for each action relative to the total evidence for all actions under consideration. This model describes the general increase in RT associated with increasing the number of choices to be considered, indicative of a global increase in the threshold for all possible outcomes (Keuken et al., 2015). Another group found that removal of the STN from the network had similar effects on choice RTs as STN deep brain stimulation in treated Parkinson's patients—selectively eliminating the delay in RT for low-probability stimuli (Antoniades et al., 2014).

The proposed thresholding and normalization functions of the STN are complementary with the Believer-Skeptic framework and can be dissociated from the hitherto-proposed role of the direct and indirect competition as a mechanism for encoding action uncertainty. The normalizing effect of STN output on pallidal inhibition emerges naturally under the assumption that all actions simultaneously engage both the direct and indirect pathways. That is, individual action uncertainty is encoded by the “short” indirect pathway from striatum to GPe and then to channel-specific populations in the GPi (see Figure 1; Schroll and Hamker, 2013) where the indirect pathway converges with action facilitating signals of the direct pathway. On the contrary, activation of the “long” indirect pathway, splitting off from GPe to the STN, leads to widespread excitatory increase in GPi firing. Under the assumption that both direct and indirect pathways are active for each action being considered, the net activation through the “long” indirect pathway has a normalizing effect on the basal GPi state, accommodating varied set sizes of alternative actions. Moreover, the relative uncertainty between actions is preserved regardless of hyper-direct perturbation of STN in the event of conflict detection. Increased hyper-direct activation of the STN would sacrifice the optimality of the normalizing constant it delivers to GPi, but only when that optimality is challenged by unanticipated conflict.

**Figure 3:**
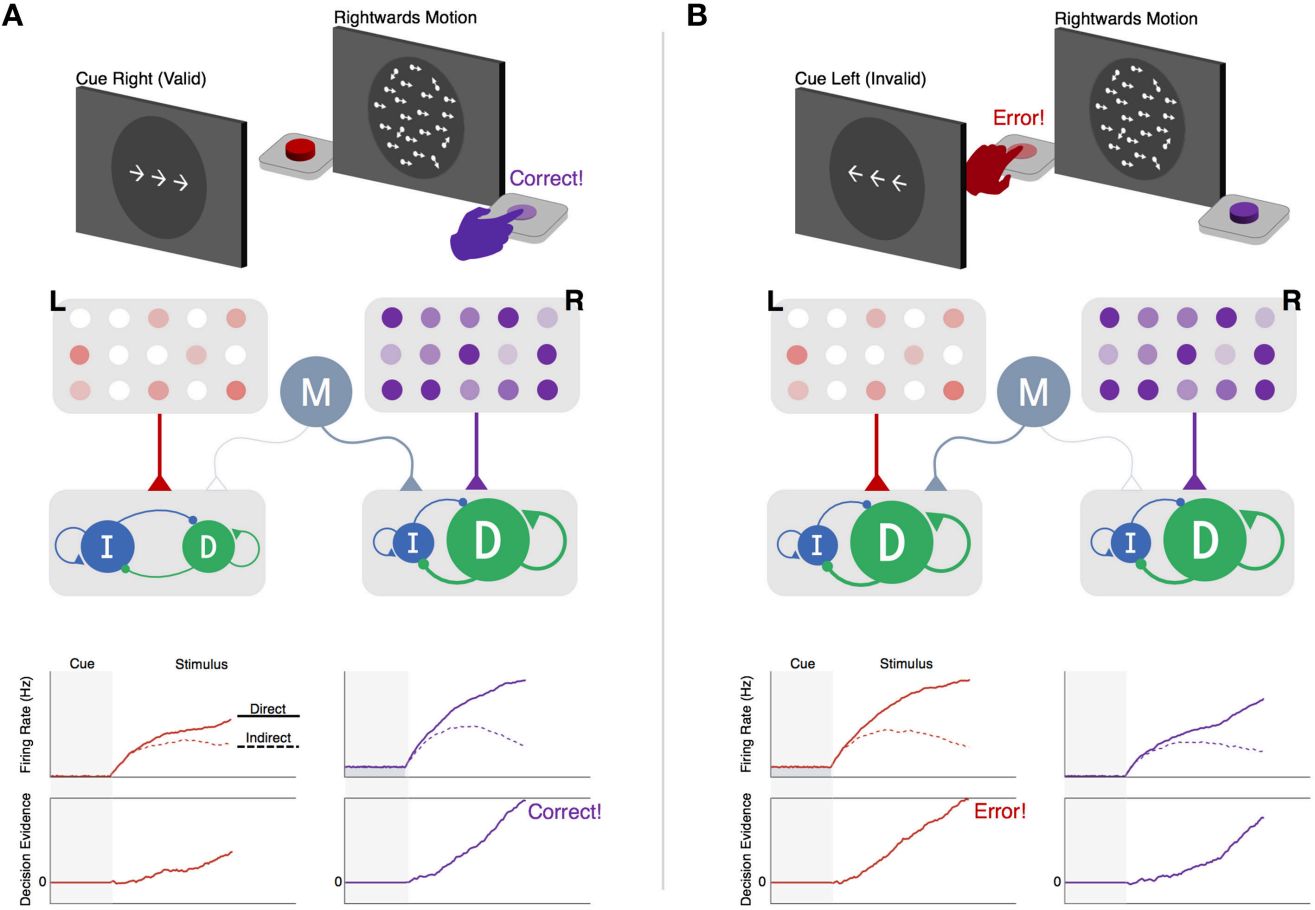
**Within-channel competition and top-down modulation of expectations during perceptual decision-making. (A) (Top)** : schematic of cue and stimulus epochs of random dot motion task on trial with valid predictive cue-stimulus combination. **(Middle)**: schematic of decision network. Left (L, red) and right (R, purple) motion-selective sensory populations gradually increase activity at a rate proportional to the strength of coherent motion in their preferred direction. Each sensory population sends excitatory input to a corresponding pair of direct and indirect populations representing left- and right-hand actions for reporting leftward and rightward motion decisions, respectively. Sensory inputs activate both pathways but with a bias favoring the direct pathway, reflecting the tendency for sensory inputs to the striatum to form more connections with dMSNs than iMSNs (Wall et al., 2013). A modulatory population (M, gray) delivers non-selective excitatory input to the pair of direct and indirect pathways encoding the anticipated action (i.e., action corresponding to the cue-predicted motion direction). **(Bottom-upper1)**: firing rates of the direct (solid line) and indirect (dotted lines) populations for left- and right-hand actions. **(Bottom-lower)**: accumulation of the difference between direct and indirect firing-rates toward an execution threshold. The effect of cued expectations can be seen as an upwards shift in the baseline firing rates of the right-hand direct-indirect network, reflecting anticipatory input from the modulatory population. This increases the excitability of the network, causing a faster separation in the direct-indirect competition and a faster rise-to-threshold in the rightwards than leftwards decision variable, producing a correct response. **(B) (Top):** same task as in the left panel but on a trial with an invalid predictive cue-stimulus combination. **(Middle)**: same decision network as in the left panel but with modulatory input delivered to the left-hand direct-indirect network as a result of cued expectations of a leftwards motion stimulus. **(Bottom)**: same layout as in the left panel. The invalid expectation signal destabilizes the direct-indirect competition, leading to a faster rise-to-threshold of the left-hand decision variable and an incorrect response.

While the long-indirect and hyper-direct pathways likely play an important role in action selection, the within-channel competition of the direct and (short) indirect pathways is ultimately what determines which action is selected. For instance, in the context of a forced-choice perceptual decision, the transition between accumulation and execution is determined by the relative activation of two alternative action channels, each driven by a separate set of competing direct and indirect populations. This process is shown in Figure 3, where an observer must decide whether a noisy field of moving dots contains greater coherent leftward or rightward motion. Critically, a cue is displayed prior to each choice informing the observer which outcome is more likely to be correct on the upcoming trial. Previous work has shown that this predictive information is encoded by a concurrent increase in the baseline activity in the striatum, contralateral to the expected action, and modulatory regions of cortex, such as orbitofrontal cortex (OFC) and pre-supplementary motor area (preSMA; Forstmann et al., 2010). When the cued probability is valid (i.e., correctly predicts the subsequent stimulus; Figure 3A) the increase in baseline activity of the corresponding action channel causes the network to become more unstable, leading to faster gating upon descending input from cortical accumulators. However, when the cue is misleading or invalid (Figure 3B), this destabilization in the cued action channel can lead to an incorrect response despite weak sensory evidence in favor of that choice. This speed-accuracy tradeoff is a widespread phenomenon that pervades all forms of decision-making. While numerous studies have found that functional and structural connectivity between preSMA and the striatum predicts individual differences in the speed-accuracy tradeoff (Forstmann et al., 2010; van Maanen et al., 2011; Keuken et al., 2014), the underlying mechanism by which modulatory cortical inputs influence action selection in the BG has remained unclear. The example here proposes one such mechanism and highlights an important prediction of the Believer-Skeptic framework, in which uncertainty associated with individual actions is encoded by the competition between corresponding direct and indirect pathways. Of course, this prediction will need to be more rigorously tested, both experimentally and through the use of more sophisticated computational models of BG circuitry.

In sum, the Believer-Skeptic framework provides a compelling account for the role of the BG in decision-making, demonstrating the computational utility for encoding action uncertainty in the competition between the direct and indirect pathways. This framework also provides a straightforward interpretation of the different roles of striatal and STN modulation of the decision process. Non-specific background inputs to the striatum can adjust the speed-accuracy tradeoff in favor of quicker decision-making by promoting faster state attraction in response to input from sensory accumulators. Cortico-striatal mechanisms may also modulate the decision in outcome-specific ways by altering the balance of channel-specific activity in the direct and indirect pathways. This interpretation is consistent with human neuroimaging studies linking cortico-striatal activity to the facilitation of one choice at the expense of choosing another; for instance, by selectively increasing of the drift-rate or baseline evidence for an expected outcome (Forstmann et al., 2010; Dunovan et al., 2015). On the other hand, indirect pathway activation of the STN provides a normalizing constant to BG output by aggregating the activation of multiple action channels into diffuse projections to the GPi, whereas hyper-direct activation of the STN modulates the decision indiscriminately, buying time in the interest of accuracy (Forstmann et al., 2012; Frank et al., 2015). In the following and final section, we elaborate on how Believer-Skeptic dynamics of decision-making are complemented by the well-established role of the cortico-striatal circuits in mediating Actor-Critic reinforcement learning (RL).

## Believer-skeptic meets Actor-Critic

The idea that direct and indirect pathway competition may be a mechanism for encoding action uncertainty has profound implications not only for decision-making, but also for reconsidering what exactly the BG learns. Feedback based learning in BG pathways has been best described as an Actor-Critic process (Sutton and Barto, 1998) where the values of alternative actions are learned by trial-and-error comparison of an action's expected and observed values. The Actor learns to select more valuable actions based on the feedback from the Critic about the difference between expected and observed rewards following an action. Thus, the critical learning signal in RL models is quantified as a reward prediction error (RPE), calculated as the difference between an action's observed and expected value. Evidence from human and animal studies has consistently linked this form of learning to phasic modulation of dopaminergic neurons in the substantia nigra pars compacta (SNc) that send feedback signals to striatal direct and indirect MSNs. When an action is followed by an unexpected reward (i.e., a positive RPE), SNc neurons display a transient burst in firing that scales with the RPE magnitude, causing proportional influx of dopamine into the striatum. In contrast, the omission of an expected reward (i.e., a negative RPE) cause a transient pause in SNc firing, thereby reducing dopamine availability in the striatum. Recent computational and experimental studies have started to build a more complete picture of the interface between controlled action decisions, as discussed in the previous section, as well as better explicate the role of dopamine in flexibly adapting goal-directed behavior. In the following section we discuss a reconceptualized model of cortico-BG pathways at the intersection of neurocomputational theories of decision-making and RL.

### Dopaminergic modulation of believer-skeptic balance

Electrophysiological studies have consistently found a relationship between the phasic activation of midbrain dopaminergic neurons and the trialwise magnitude of RPEs that mediate RL. For this dopaminergic RPE to be a viable learning signal it must be capable of selectively encouraging rewarded actions and discouraging unrewarded or punished actions. The phasic increase in dopamine following a surprising reward both sensitizes dMSNs and desensitizes iMSNs, making it easier for cortical inputs to quickly execute that action in the future (Wiecki and Frank, 2013; Hart et al., 2014). By the same token, phasic dips in dopamine following the omission of an expected reward offset the balance in the other direction, requiring stronger or prolonged cortical input to gate the same action in the future (Marcott et al., 2014; Bahuguna et al., 2015; Gurney et al., 2015). The bidirectional effect of positive and negative feedback on pathway-specific neural subtypes sheds light on the utility of selecting actions with two opposing pathways instead of a single facilitation pathway (Hart et al., 2014). Indeed, several lines of evidence suggest that dopaminergic modulation of the direct pathway is primarily driven by positive RPEs that facilitate approach-learning, whereas the modulation of the indirect pathway is primarily driven by negative RPEs, facilitating avoidance learning (Frank et al., 2009; Hikida et al., 2013; Cox et al., 2015).

In a series of computational experiments, Gurney et al. (2015) recently provided a comprehensive description of the interactions between tonic and phasic fluctuations in striatal dopamine that guide goal-directed action selection. In their neural network model, cortical input from competing sensory populations is sent in parallel to all three cortico-BG pathways representing the sensory-paired actions. Thus, when sensory information is equivocal and cortical input leads to comparable activation in different action channels, the history dependent cortico-striatal weights are what critically determine which of the two actions wins out in the selection process.

**Figure 4:**
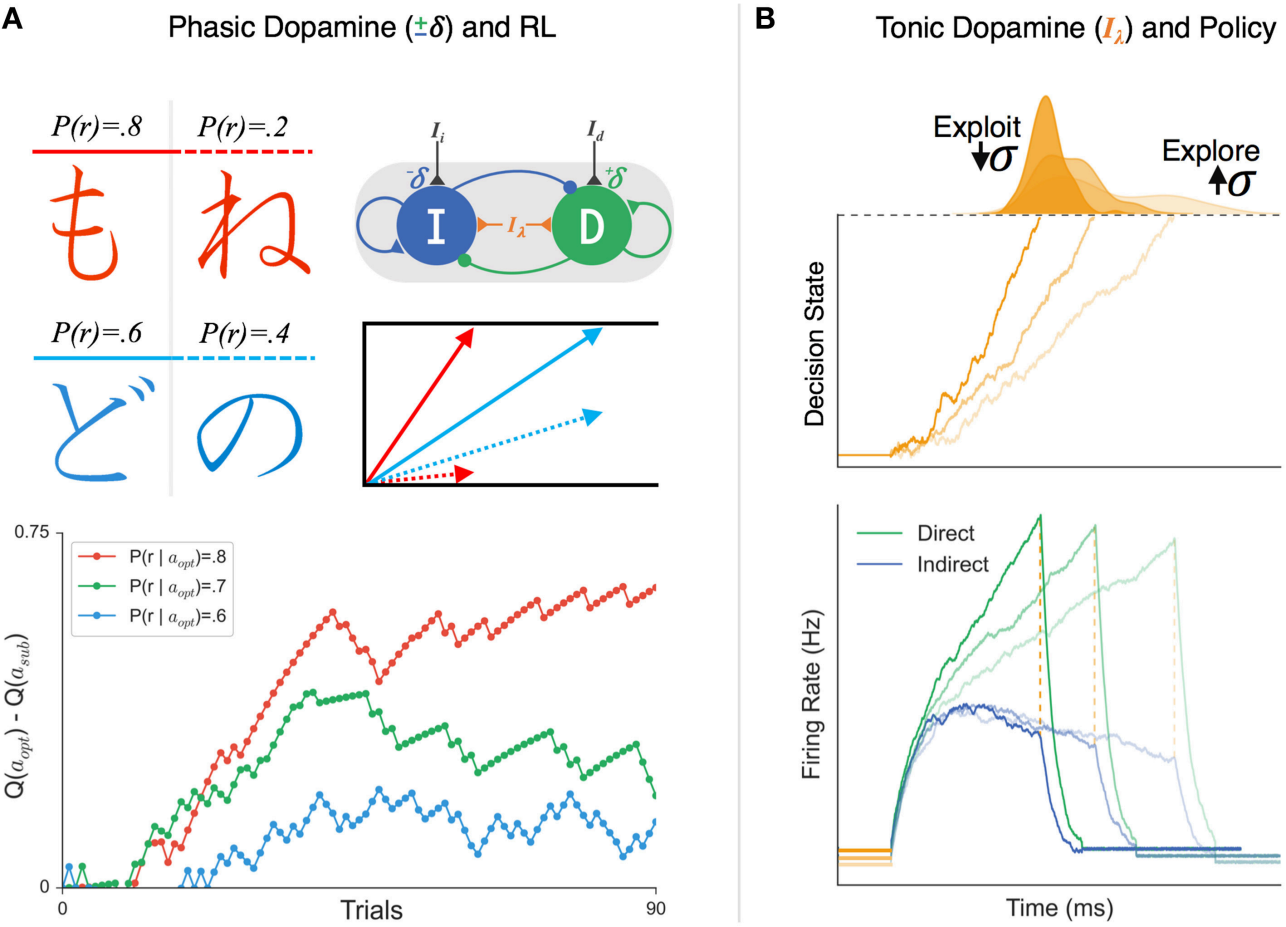
**The effects of phasic and tonic dopamine on Believer-Skeptic competition. (A)** Simulation of probabilistic value-based decision task (upper-left; see Frank et al., 2004) in which the agent must learn the relative value of two arbitrary stimuli based on trial-and-error feedback. On each trial the agent makes a decision by choosing between a pair of Japanese symbols, one with a higher probability of yielding a reward (left column; chosen with action *a*_*opt*_) than the other (right column; chosen with action *a*_*sub*_). Value-based decisions are simulated as a race-to-threshold between two stochastic accumulators (see Figure 3), each reflecting the direct-indirect competition within a single action channel (see Figure 2). Both actions start out with equal associated values Q(*a*_*opt*_) = Q(*a*_*sub*_) and thus, equal drift-rates of accumulation. On each trial, the corrective effects of phasic changes in dopamine are simulated by enhancing (depressing) the sensitivity of the direct (indirect) pathway following positive outcomes (+δ) and vice-versa following negative outcomes (−δ). In the accumulator model, this learning results in an increase in the drift-rate for *a*_*opt*_ (solid arrow) and a decrease in the drift-rate for *a*_*sub*_ (dotted arrow), proportional to the difference in their associated value. The bottom panel shows the timeline of the estimated value difference for alternative actions (Q(*a*_*opt*_) − Q(*a*_*sub*_)) for three different probabilistic reward schedules. Stimulus pairs with a greater discrepancy in reward probability (i.e., red > green > blue) lead to faster associative value learning. **(B)** Simulated effects of tonic dopamine levels on exploration-exploitation tradeoff. Tonic dopamine levels were simulated by varying the strength of non-specific background inputs (I_λ_) in a network with stronger weighting of cortical input to direct than indirect pathway. (**Bottom**) panel: the same ratio of cortical input to the direct (green) and indirect (blue) pathways leads to faster gating in the presence higher I_λ_ (darker colors, increased baseline) compare to when I_λ_ is low (lighter colors, decreased baseline). (**Top**) panel: Increasing tonic levels of I_λ_ facilitates exploitation of the current cortico-striatal weights by accelerating evidence accumulation, resulting in faster decisions and reduced trial-to-trial variability in RT. In contrast, behavior is substantially more variable with lower levels of I_λ_, promoting an exploration policy.

The synaptic tuning of these weights by positive and negative RPEs can be naturally incorporated into the Believer-Skeptic decision network shown in Figure 2A—by increasing the sensitivity of the direct and indirect populations following rewarded and punished actions, respectively. Over the course of several trials, the feedback-dependent tuning of synaptic weights leads to faster gating in the network and thus faster rates of evidence accumulation in decision space for higher valued actions. This is captured in Figure 4A where the model gradually learns the relative value of alternative actions based on probabilistic stimulus-reward contingencies from trial-and-error feedback. Similar to the behavioral paradigm used by Frank et al. (2004) the model is presented with a pair of stimuli and must learn to select the stimulus with a higher probability of yielding a reward. Each stimulus is converted into an action by a corresponding pair of direct and indirect nodes that are tuned by corrective feedback signals, simulating the effects of dopaminergic RPE signals on dMSNs and iMSNs. Thus, feedback sensitizes the direct pathway and suppresses the indirect pathway for the optimal choice while shifting the balance in the opposite direction for the alternative, converging on weights that reflect the expected difference in their learned values. In the accumulator model, this manifests as a drift-rate for each stimulus proportional to its perceived value, leading to a stronger choice bias when deciding between alternatives that are less evenly matched in terms of their expected payout (Figure 4A).

Because in this example the stimulus-action-value associations are probabilistic, a certain amount of exploration is needed in order to optimize the estimated value for each of the two stimuli. In Actor-Critic RL, exploratory dynamics are usually facilitated by a single parameter that determines the initial probability of going with the currently highest-valued option. Here, however, exploration is naturally handled by the stochastic nature of the direct-indirect competition during the decision process. A recent study found that the RT distributions of value-based choices in a perceptual learning experiment were well described by a DDM in which the learned value difference between alternative stimuli determined the drift-rate of accumulation (Frank et al., 2015). This finding adds support to the future hybridization of RL and decision models, suggesting that the behavioral dynamics of value-based choices can be systematically characterized by corrective modulation of a stochastic rise-to-threshold process.

In addition to the phasic dopamine modulations responsible for learning action-value associations, the level of tonic dopamine availability in the striatum has recently been proposed to regulate the tradeoff between exploratory and exploitative learning policies (Humphries et al., 2012; Kayser et al., 2015). That is, in order to maximize rewards in dynamic environments (with changing response-outcome contingencies), one must balance the time spent exploring the value of novel, potentially high-payoff actions, and exploiting historically rewarding actions (Humphries et al., 2012; Keeler et al., 2014). Put into the context of the Believer-Skeptic framework, explorative states can be thought of as conditions in which the balance is tipped toward the Skeptic such that all action possibilities are uncertain and thus no single decision dominates. In contrast, exploitative states are those in which the Believer dominates for a single decision, resulting in faster and more precise decisions that preclude alternative actions from being engaged.

Much of the current understanding of the interplay between value-based learning mechanisms and exploitation-exploration tradeoff policies has come from research on song-bird learning (Brainard and Doupe, 2002; Kao et al., 2005). While research on song-bird learning has progressed largely in parallel with the studies of decision-making in the BG, it has been speculated that the two fields are currently moving toward a mutually beneficial junction (Ding and Perkel, 2014). Juvenile song-birds initially learn to sing by mirroring the song of an experienced tutor but over time compose an individualized version of the song by sampling alternate spectral and temporal components of vocalization (Tumer and Brainard, 2007). This is done to improve reproductive success, as females tend to select males with unique songs that can be performed repeatedly with high precision. Recently Woolley et al. (2014) found that when practicing in isolation, males express substantially more variability in the spectral and temporal dimensions of song vocalization than when in the presence of a mate. This contextual alternation between exploring alternate song renditions during practice and exploiting a favorite rendition led to systematic differences in the variability of firing in the output of a region called Area X, a homolog of the mammalian BG. The authors proposed that social context led to changes in the tonic level of dopamine available to neurons in the input structure of Area X, similar to the striatum of the BG in mammals, which impacted the amount of exploration or exploitation of the system. Their hypothesis was supported by the observation that striatal connections exhibit a many-to-one convergence onto target cells in the BG output nucleus. Previous work suggests that given this many-to-one motif, enhanced dopaminergic tone would establish a more consistent average level of activation within a group of striatal units, thus increasing reliability of temporally-locked bursts and pauses of recipient neurons in the output nucleus (Goldberg et al., 2004; Costa et al., 2006).

Consistent with a dopaminergic regulation between exploitative-explorative policies, several recent computational modeling studies have found that the simulated effects of tonic dopamine level have a marked impact on action variability (Yawata et al., 2012; Klanker et al., 2013; Morita and Kato, 2014). Increasing dopaminergic availability in the striatum leads to a general “Go” bias in the network, due to the inverse effects of dopamine on MSN subpopulations. Furthermore, higher tonic dopamine levels also increases D1 and D2 receptor occupancy so that RPE signals communicated by phasic bursts and pauses in SNc fail to have the same impact on cortico-striatal plasticity (Keeler et al., 2014). Thus, behavior is stabilized to promote exploitation of previously learned associations by facilitating BG throughput that reflects the present weighting scheme at cortico-striatal synapses. In Figure 4B, the population firing rates are shown for different decision policies, all reflecting the same ratio of input to the direct and indirect pathways, but with a change in background levels of tonic dopamine (e.g., background excitation). Increasing dopamine reduces the time constant of evidence accumulation such that learned cortico-striatal weights can be exploited to rapidly accelerate the network toward a “Go” state, with little variability in the RT and outcome of the decision process (Figure 4B). Alternatively, the same levels of cortical input leads to substantially greater trial-to-trial variability in decision behavior when dopamine is scarce, demonstrated by the widening of the RT distribution for decisions made under lower levels of background dopamine. When considered in the context of selecting from multiple actions, the increase in action variability (i.e., wider RT distribution) with reduced levels of tonic dopamine would allow the agent to explore novel, potentially more rewarding, stimulus-action associations. When a sufficiently rewarding association is found or when there is a change in context that demands precision, increasing background dopamine levels would temporarily halt feedback-dependent plasticity to ensure lower variability in performance.

The relationship between action variability and striatal dopamine adds an interesting perspective to recent studies showing how behavioral variability expands and contracts with a subject's learning rate, and seems to do so in a controlled, systematic fashion. While standard RL models assume learning rate to be a constant index of an individual's inherent sensitivity to feedback error, applying this assumption to human behavior seems to be overly restrictive, especially in realistically dynamic environments. It has been hypothesized, that in settings with a high probability of experiencing a state change (i.e., change in a previously learned stimulus-response-outcome mappings) humans may deliberately amplify the uncertainty or perceived risk of their surroundings so as to maximize adaptability to new information (O'Reilly, 2013). Indeed, a recent study by Wu et al. (2014) found that when learning to make visually-guided and reward-guided reaching movements, human subjects demonstrated a simultaneous increase in learning rate with movement variability during times of greater uncertainty. Incredibly, the authors found that the increase in motor variability was not random, but was expressed along task-specific dimensions, suggesting that variability is not only capitalized on but is deliberately employed by the nervous system to facilitate adaptation to relevant sources of error. In addition to continuous motor control experiments like this one, discrete choice experiments have found that variability in decision-making strategically fluctuates with model-fit learning rates in response to a shift in the statistics of a task or environment (Nassar et al., 2012; Payzan-LeNestour et al., 2013; McGuire et al., 2014). Franklin and Frank (2015) recently proposed a candidate mechanism for adapting the rate of learning to changes in uncertainty based on the modulatory influence of cholinergic interneurons on striatal plasticity in BG model of decision making. These tonically active (inter)neurons (TANs) are known to exhibit a pause in firing in response to reinforcement feedback following action execution. This pause in TAN activity results in disinhibition of both dMSNs and iMSNs. Because this pause is temporally concomitant with dopaminergic RPEs, the resulting disinhibition of MSNs improves activity-dependent plasticity and, in turn, the divergence of synaptic weighting in optimal **(**↑direct, ↓indirect) and suboptimal **(**↓direct,↑indirect) channels. A novel prediction of the model is that uncertainty in the decision process can be estimated online based on the entropy of MSN activity across action channels and over time. The authors show that, under certain circumstances, the reciprocal connectivity between striatal MSNs and TANs is capable of dynamically adapting the learning rate to optimize the exploration-exploitation tradeoff across varying degrees of uncertainty. For instance, high entropic MSN activity leads to longer TAN pauses, enhancing plasticity in the context of high uncertainty (i.e., following rule reversal) to accelerate acquisition of new associations. Future electrophysiological studies will be needed to confirm the prediction that decision uncertainty is somehow encoded by the firing activity of MSNs across alternative action channels and that this population code plays a role in optimizing plasticity during RL.

Human neuroimaging studies have also found evidence of striatal involvement in learning rate adaptation. For instance, a recent fMRI study found that activity in the caudate nucleus dynamically tracks subjects' learning rates, rising with greater trial-wise volatility in choice difficulty across blocks of a Stroop task (Jiang et al., 2015). The authors found that the volatility-driven changes in caudate activation resulted from descending control signals in the anterior cingulate cortex (ACC), updating the predicted level of control needed on the upcoming trial. One intriguing explanation for this finding is that exploratory dynamics in the striatum are mediated by different sources depending on the dimension of exploration: i.e., relying on modulatory cortical inputs to facilitate control-based exploration and tonic dopamine levels to facilitate value exploration (Woolley et al., 2014). Of course, this mechanism is only speculative and future studies investigating the mechanisms of control- and value-based exploration will need to draw on evidence from both animal models as well as human neuroimaging experiments. Neuroimaging experiments in particular could be poised to investigate this question by comparing the functional connectivity between cortical regions, such as ACC and preSMA, and the striatum across conditions in which task performance relies on state-change detection of stimulus-control or stimulus-value associations. Furthermore, pharmacological manipulations could be employed to determine if value-based exploration is selectively impaired by increasing tonic dopamine availability.

## Summary and conclusions

The emerging evidence on the organization of and interactions between BG pathways highlights the limitations of the canonical model of parallel and independent pathways. While the canonical model continues to provide a valuable benchmark for evaluating advancements in the understanding BG function, recent evidence suggests that competition between BG pathways has profound implications for understanding the BG's role in decision making and learning. Here, we have presented an overview of recent experimental and computational evidence for a reconceptualized view of cortico-BG pathways, highlighting three central themes: (1) the direct and indirect pathways engage in competition during action selection, acting as weights on a pulley, rather than independent facilitation and suppression levers, (2) this competition is critical for integrating contextual uncertainty (i.e., Skeptic) with accumulating evidence (i.e., Believer) during decision making, and (3) this competitive dynamic lays the foundation for a rich, flexible behavioral repertoire when combined with the dopaminergic modulation described by Actor-Critic RL theories. Based on these findings we have outlined a conceptual framework for the decision-making computations embedded in the competition between the direct and indirect pathways. We feel that this Believer-Skeptic framework offers an appealing first step toward synthesizing neurocomputational theories of decision making with Actor-Critic models of RL.

## Author contributions

KD and TV made equal intellectual contributions to content of the manuscript. KD wrote the code for the model simulations and created the figures.

### Conflict of interest statement

The authors declare that the research was conducted in the absence of any commercial or financial relationships that could be construed as a potential conflict of interest.

## References

[B1] Albin R. L. Young A. B. Penney J. B. Roger L. A. Young B. B. (1989). The functional anatomy of basal ganglia disorders. Trends Neurosci. 12, 366–375. 10.1016/0166-2236(89)90074-X 2479133

[B2] Alexander G. E. Crutcher M. D. (1990). Functional architecture of basal ganglia circuitry: neural substrates of parallel processing. Trends Neurosci. 13, 266–271. 10.1016/0166-2236(90)90107-L 1695401

[B3] Antoniades C. A. Bogacz R. Kennard C. FitzGerald J. J. Aziz T. Green A. L. (2014). Deep brain stimulation abolishes slowing of reactions to unlikely stimuli. J. Neurosci. 34, 10844–10852. 10.1523/JNEUROSCI.1065-14.2014 25122887 PMC4131008

[B4] Aron A. R. Poldrack R. A. (2006). Cortical and subcortical contributions to Stop signal response inhibition: role of the subthalamic nucleus. J. Neurosci. 26, 2424–2433. 10.1523/JNEUROSCI.4682-05.2006 16510720 PMC6793670

[B5] Averbeck B. B. Lehman J. Jacobson M. Haber S. N. (2014). Estimates of projection overlap and zones of convergence within frontal-striatal circuits. J. Neurosci. 34, 9497–9505. 10.1523/JNEUROSCI.5806-12.2014 25031393 PMC4099536

[B6] Bahuguna J. Aertsen A. Kumar A. (2015). Existence and control of go/no-go decision transition threshold in the striatum. PLoS Comput. Biol. 11:e1004233. 10.1371/journal.pcbi.1004233 25910230 PMC4409064

[B7] Bogacz R. Gurney K. (2007). The basal ganglia and cortex implement optimal decision making between alternative actions. Neural Comput. 19, 442–477. 10.1162/neco.2007.19.2.442 17206871

[B8] Bogacz R. Wagenmakers E. J. Forstmann B. U. Nieuwenhuis S. (2010). The neural basis of the speed-accuracy tradeoff. Trends Neurosci. 33, 10–16. 10.1016/j.tins.2009.09.002 19819033

[B9] Brainard M. S. Doupe A. J. (2002). What songbirds teach us about learning. Nature 417, 351–358. 10.1038/417351a 12015616

[B10] Braver T. S. (2012). The variable nature of cognitive control: a dual mechanisms framework. Trends Cogn. Sci. 16, 106–113. 10.1016/j.tics.2011.12.010 22245618 PMC3289517

[B11] Brown J. W. Bullock D. Grossberg S. (2004). How laminar frontal cortex and basal ganglia circuits interact to control planned and reactive saccades. Neural Netw. 17, 471–510. 10.1016/j.neunet.2003.08.006 15109680

[B12] Calabresi P. Picconi B. Tozzi A. Ghiglieri V. Di Filippo M. (2014). Direct and indirect pathways of basal ganglia: a critical reappraisal. Nat. Neurosci. 17, 1022–1030. 10.1038/nn.3743 25065439

[B13] Cavanagh J. F. Wiecki T. V. Cohen M. X. Figueroa C. M. Samanta J. Sherman S. J.. (2011). Subthalamic nucleus stimulation reverses mediofrontal influence over decision threshold. Nat. Neurosci. 14, 1462–1467. 10.1038/nn.2925 21946325 PMC3394226

[B14] Cazorla M. de Carvalho F. D. Chohan M. O. Shegda M. Chuhma N. Rayport S.. (2014). Dopamine D2 receptors regulate the anatomical and functional balance of basal ganglia circuitry. Neuron 81, 153–164. 10.1016/j.neuron.2013.10.041 24411738 PMC3899717

[B15] Chikazoe J. Jimura K. Hirose S. Yamashita K. Miyashita Y. Konishi S. (2009). Preparation to inhibit a response complements response inhibition during performance of a stop-signal task. J. Neurosci. 29, 15870–15877. 10.1523/JNEUROSCI.3645-09.2009 20016103 PMC6666181

[B16] Costa R. M. Lin S. C. Sotnikova T. D. Cyr M. Gainetdinov R. R. Caron M. G.. (2006). Rapid alterations in corticostriatal ensemble coordination during acute dopamine-dependent motor dysfunction. Neuron 52, 359–369. 10.1016/j.neuron.2006.07.030 17046697

[B17] Cox S. M. L. Frank M. J. Larcher K. Fellows L. K. Clark C. A. Leyton M.. (2015). Striatal D1 and D2 signaling differentially predict learning from positive and negative outcomes. Neuroimage 109, 95–101. 10.1016/j.neuroimage.2014.12.070 25562824

[B18] Cui G. Jun S. B. Jin X. Pham M. D. Vogel S. S. Lovinger D. M.. (2013). Concurrent activation of striatal direct and indirect pathways during action initiation. Nature 494, 238–242. 10.1038/nature11846 23354054 PMC4039389

[B19] DeLong M. R. (1990). Primate models of movement disorders of basal ganglia origin. Trends Neurosci. 13, 281–285. 10.1016/0166-2236(90)90110-V 1695404

[B20] Ding L. Gold J. I. (2010). Caudate encodes multiple computations for perceptual decisions. J. Neurosci. 30, 15747–15759. 10.1523/JNEUROSCI.2894-10.2010 21106814 PMC3005761

[B21] Ding L. Gold J. I. (2012a). Neural correlates of perceptual decision making before, during, and after decision commitment in monkey frontal eye field. Cereb. Cortex 22, 1052–1067. 10.1093/cercor/bhr178 21765183 PMC3328342

[B22] Ding L. Gold J. I. (2012b). Separate, causal roles of the caudate in saccadic choice and execution in a perceptual decision task. Neuron 75, 865–874. 10.1016/j.neuron.2012.07.021 22958826 PMC3446771

[B23] Ding L. Gold J. I. (2013). The basal ganglia's contributions to perceptual decision making. Neuron 79, 640–649. 10.1016/j.neuron.2013.07.042 23972593 PMC3771079

[B24] Ding L. Perkel D. J. (2014). Two tales of how expectation of reward modulates behavior. Curr. Opin. Neurobiol. 29, 142–147. 10.1016/j.conb.2014.07.011 25062505 PMC4254302

[B25] Dodson P. D. D. Larvin J. T. T. Duffell J. M. M. Garas F. N. N. Doig N. M. M. Kessaris N.. (2015). Distinct developmental origins manifest in the specialized encoding of movement by adult neurons of the external globus pallidus. Neuron 86, 1–13. 10.1016/j.neuron.2015.03.007 25843402 PMC4416107

[B26] Draganski B. Kherif F. Klöppel S. Cook P. A. Alexander D. C. Parker G. J. M.. (2008). Evidence for segregated and integrative connectivity patterns in the human Basal Ganglia. J. Neurosci. 28, 7143–7152. 10.1523/JNEUROSCI.1486-08.2008 18614684 PMC6670486

[B27] Dunovan K. Lynch B. Molesworth T. Verstynen T. (2015). Competing basal ganglia pathways determine the difference between stopping and deciding not to go. eLife 4:e08723. 10.7554/elife.08723 26402462 PMC4686424

[B28] Forstmann B. U. Brown S. Dutilh G. Neumann J. Wagenmakers E.-J. (2010). The neural substrate of prior information in perceptual decision making: a model-based analysis. Front. Hum. Neurosci. 4:40. 10.3389/fnhum.2010.00040 20577592 PMC2889713

[B29] Forstmann B. U. Dutilh G. Brown S. Neumann J. von Cramon D. Y. Ridderinkhof K. R.. (2008). Striatum and pre-SMA facilitate decision-making under time pressure. Proc. Natl. Acad. Sci. U.S.A. 105, 17538–17542. 10.1073/pnas.0805903105 18981414 PMC2582260

[B30] Forstmann B. U. Keuken M. C. Jahfari S. Bazin P.-L. Neumann J. Schäfer A.. (2012). Cortico-subthalamic white matter tract strength predicts interindividual efficacy in stopping a motor response. Neuroimage 60, 370–375. 10.1016/j.neuroimage.2011.12.044 22227131

[B31] Frank M. J. Doll B. B. Oas-terpstra J. Moreno F. (2009). Prefrontal and striatal dopaminergic genes predict individual differences in exploration and exploitation. Nat. Neurosci. 12, 1062–1068. 10.1038/nn.2342 19620978 PMC3062477

[B32] Frank M. J. Gagne C. Nyhus E. Masters S. Wiecki T. V. Cavanagh X. F.. (2015). fMRI and EEG predictors of dynamic decision parameters during human reinforcement learning. J. Neurosci. 35, 485–494. 10.1523/JNEUROSCI.2036-14.2015 25589744 PMC4293405

[B33] Frank M. J. Seeberger L. C. O'Reilly R. C. (2004). By carrot or by stick: cognitive reinforcement learning in Parkinsonism. Science 306, 1940–1943. 10.1126/science.1102941 15528409

[B34] Franklin N. T. Frank M. J. (2015). A cholinergic feedback circuit to regulate striatal population uncertainty and optimize reinforcement learning. eLife 4:e12029. 10.7554/eLife.12029 26705698 PMC4764588

[B35] Freeze B. S. Kravitz A. V. Hammack N. Berke J. D. Kreitzer A. C. (2013). Control of basal ganglia output by direct and indirect pathway projection neurons. J. Neurosci. 33, 18531–18539. 10.1523/JNEUROSCI.1278-13.2013 24259575 PMC3834057

[B36] Friend D. M. Kravitz A. V. (2014). Working together: basal ganglia pathways in action selection. Trends Neurosci. 37, 301–303. 10.1016/j.tins.2014.04.004 24816402 PMC4041812

[B37] Goldberg J. A. Rokni U. Boraud T. Vaadia E. Bergman H. (2004). Spike synchronization in the cortex-basal ganglia networks of Parkinsonian primates reflects global dynamics of the local field potentials. J. Neurosci. 24, 6003–6010. 10.1523/JNEUROSCI.4848-03.2004 15229247 PMC6729228

[B38] Gurney K. N. Humphries M. D. Redgrave P. (2015). A new framework for cortico-striatal plasticity: behavioural theory meets *in vitro* data at the reinforcement-action interface. PLoS Biol. 13:e1002034. 10.1371/journal.pbio.1002034 25562526 PMC4285402

[B39] Haber S. Kunishio K. Mizobuchi M. Lynd-Balta E. (1995). The orbital and medial prefrontal circuit through the primate basal ganglia. J. Neurosci. 15, 4851–4867. 7623116 10.1523/JNEUROSCI.15-07-04851.1995 PMC6577885

[B40] Haber S. N. (2014). The place of dopamine in the cortico-basal ganglia circuit. Neuroscience 282, 248–257. 10.1016/j.neuroscience.2014.10.008 25445194 PMC5484174

[B41] Hart A. S. Rutledge R. B. Glimcher P. W. Phillips P. E. M. (2014). Phasic dopamine release in the rat nucleus accumbens symmetrically encodes a reward prediction error term. J. Neurosci. 34, 698–704. 10.1523/JNEUROSCI.2489-13.2014 24431428 PMC3891951

[B42] Haynes W. I. A. Haber S. N. (2013). The organization of prefrontal-subthalamic inputs in primates provides an anatomical substrate for both functional specificity and integration: implications for Basal Ganglia models and deep brain stimulation. J. Neurosci. 33, 4804–4814. 10.1523/JNEUROSCI.4674-12.2013 23486951 PMC3755746

[B43] Heitz R. P. Schall J. D. (2012). Neural mechanisms of speed-accuracy tradeoff. Neuron 76, 616–628. 10.1016/j.neuron.2012.08.030 23141072 PMC3576837

[B44] Heitz R. P. Schall J. D. (2013). Neural chronometry and coherency across speed-accuracy demands reveal lack of homomorphism between computational and neural mechanisms of evidence accumulation. Philos. Trans. R. Soc. Lond. B Biol. Sci. 368:20130071. 10.1098/rstb.2013.0071 24018731 PMC3758212

[B45] Hikida T. Kimura K. Wada N. Funabiki K. Nakanishi S. (2010). Distinct roles of synaptic transmission in direct and indirect striatal pathways to reward and aversive behavior. Neuron 66, 896–907. 10.1016/j.neuron.2010.05.011 20620875

[B46] Hikida T. Yawata S. Yamaguchi T. Danjo T. Sasaoka T. Wang Y.. (2013). Pathway-specific modulation of nucleus accumbens in reward and aversive behavior via selective transmitter receptors. Proc. Natl. Acad. Sci. U.S.A. 110, 342–347. 10.1073/pnas.1220358110 23248274 PMC3538201

[B47] Huerta-Ocampo I. Mena-Segovia J. Bolam J. P. (2013). Convergence of cortical and thalamic input to direct and indirect pathway medium spiny neurons in the striatum. Brain Struct. Funct. 2, 1–14. 10.1007/s00429-013-0601-z PMC4147250 23832596

[B48] Humphries M. D. Khamassi M. Gurney K. (2012). Dopaminergic control of the exploration-exploitation trade-off via the basal ganglia. Front. Neurosci. 6:9. 10.3389/fnins.2012.00009 22347155 PMC3272648

[B49] Jahfari S. Stinear C. M. Claffey M. Verbruggen F. Aron A. R. (2010). Responding with restraint: what are the neurocognitive mechanisms? J. Cogn. Neurosci. 22, 1479–1492. 10.1162/jocn.2009.21307 19583473 PMC2952035

[B50] Jahfari S. Verbruggen F. Frank M. J. Waldorp L. Colzato L. Ridderinkhof K. R.. (2012). How preparation changes the need for top-down control of the basal ganglia when inhibiting premature actions. J. Neurosci. 32, 10870–10878. 10.1523/JNEUROSCI.0902-12.2012 22875921 PMC6621019

[B51] Jahfari S. Waldorp L. van den Wildenberg W. P. M. Scholte H. S. Ridderinkhof K. R. Forstmann B. U. (2011). Effective connectivity reveals important roles for both the hyperdirect (fronto-subthalamic) and the indirect (fronto-striatal-pallidal) fronto-basal ganglia pathways during response inhibition. J. Neurosci. 31, 6891–6899. 10.1523/JNEUROSCI.5253-10.2011 21543619 PMC6632844

[B52] Jarbo K. Verstynen T. D. (2015). Converging structural and functional connectivity of orbitofrontal, dorsolateral prefrontal, and posterior parietal cortex in the human striatum. J. Neurosci. 35, 3865–3878. 10.1523/JNEUROSCI.2636-14.2015 25740516 PMC4461697

[B53] Jiang J. Beck J. Heller K. Egner T. (2015). An insula-frontostriatal network mediates flexible cognitive control by adaptively predicting changing control demands. Nat. Commun. 6, 1–11. 10.1038/ncomms9165 26391305 PMC4595591

[B54] Kao M. H. Doupe A. J. Brainard M. S. (2005). Contributions of an avian basal ganglia-forebrain circuit to real-time modulation of song. Nature 433, 638–643. 10.1038/nature03127 15703748

[B55] Kayser A. S. Mitchell J. M. Weinstein D. Frank M. J. (2015). Dopamine, locus of control, and the exploration-exploitation tradeoff. Neuropsychopharmacology 40, 454–462. 10.1038/npp.2014.193 25074639 PMC4443960

[B56] Keeler J. F. Pretsell D. O. Robbins T. W. (2014). Functional implications of dopamine D1 vs D2 receptors: a “Prepare and Select” model of the striatal direct vs. indirect pathways. Neuroscience 282, 156–175. 10.1016/j.neuroscience.2014.07.021 25062777

[B57] Keuken M. C. Langner R. Eickhoff S. B. Forstmann B. U. Neumann J. (2014). Brain networks of perceptual decision-making: an fMRI ALE meta-analysis. Front. Hum. Neurosci. 8:445. 10.3389/fnhum.2014.00445 24994979 PMC4063192

[B58] Keuken M. C. Van Maanen L. Bogacz R. Schäfer A. Neumann J. Turner R.. (2015). The subthalamic nucleus during decision-making with multiple alternatives. Hum. Brain Mapp. 36, 4041–4052. 10.1002/hbm.22896 26178078 PMC4896390

[B59] King A. V. Linke J. Gass A. Hennerici M. G. Tost H. Poupon C.. (2012). Microstructure of a three-way anatomical network predicts individual differences in response inhibition: a tractography study. Neuroimage 59, 1949–1959. 10.1016/j.neuroimage.2011.09.008 21939775

[B60] Klanker M. Feenstra M. Denys D. (2013). Dopaminergic control of cognitive flexibility in humans and animals. Front. Neurosci. 7:201. 10.3389/fnins.2013.00201 24204329 PMC3817373

[B61] Kravitz A. V. Freeze B. S. Parker P. R. L. Kay K. Thwin M. T. Deisseroth K.. (2010). Regulation of parkinsonian motor behaviours by optogenetic control of basal ganglia circuitry. Nature 466, 622–626. 10.1038/nature09159 20613723 PMC3552484

[B62] Kress G. J. Yamawaki N. Wokosin D. L. Wickersham I. R. Shepherd G. M. G. Surmeier D. J. (2013). Convergent cortical innervation of striatal projection neurons. Nat. Neurosci. 16, 665–667. 10.1038/nn.3397 23666180 PMC4085670

[B63] Lo C.-C. Wang X.-J. (2006). Cortico-basal ganglia circuit mechanism for a decision threshold in reaction time tasks. Nat. Neurosci. 9, 956–963. 10.1038/nn1722 16767089

[B64] Majid D. S. A. Cai W. Corey-Bloom J. Aron A. R. (2013). Proactive selective response suppression is implemented via the basal ganglia. J. Neurosci. 33, 13259–13269. 10.1523/JNEUROSCI.5651-12.2013 23946385 PMC3742918

[B65] Mallet N. Micklem B. R. Henny P. Brown M. T. Williams C. Bolam J. P.. (2012). Dichotomous organization of the external globus pallidus. Neuron 74, 1075–1086. 10.1016/j.neuron.2012.04.027 22726837 PMC3407962

[B66] Mallet N. Schmidt R. Leventhal D. Chen F. Amer N. Boraud T. (2016). Arkypallidal cells send a stop signal to striatum. Neuron 89, 308–316. 10.1016/j.neuron.2015.12.017 PMC4871723 26777273

[B67] Mansfield E. L. Karayanidis F. Jamadar S. Heathcote A. Forstmann B. U. (2011). Adjustments of response threshold during task switching: a model-based functional magnetic resonance imaging study. J. Neurosci. 31, 14688–14692. 10.1523/JNEUROSCI.2390-11.2011 21994385 PMC6703389

[B68] Marcott P. F. Mamaligas A. A. Ford C. P. (2014). Phasic dopamine release drives rapid activation of striatal D2-receptors. Neuron 84, 164–176. 10.1016/j.neuron.2014.08.058 25242218 PMC4325987

[B69] Mastro K. J. Bouchard R. S. Holt H. A. K. Gittis A. H. (2014). Transgenic mouse lines subdivide external segment of the globus pallidus (GPe) neurons and reveal distinct GPe output pathways. J. Neurosci. 34, 2087–2099. 10.1523/JNEUROSCI.4646-13.2014 24501350 PMC3913864

[B70] Mathai A. Smith Y. (2011). The corticostriatal and corticosubthalamic pathways: two entries, one target. so what? Front. Syst. Neurosci. 5, 1–10. 10.3389/fnsys.2011.00064 21866224 PMC3149683

[B71] McGuire J. T. T. Nassar M. R. R. Gold J. I. I. Kable J. W. W. (2014). Functionally dissociable influences on learning rate in a dynamic environment. Neuron 84, 870–881. 10.1016/j.neuron.2014.10.013 25459409 PMC4437663

[B72] Mink J. W. (1996). The basal ganglia: focused selection and inhibition of competing motor programs. Progr. Neurobiol. 50, 381–425. 10.1016/S0301-0082(96)00042-1 9004351

[B73] Morita K. Kato A. (2014). Striatal dopamine ramping may indicate flexible reinforcement learning with forgetting in the cortico-basal ganglia circuits. Front. Neural Circuits 8:36. 10.3389/fncir.2014.00036 24782717 PMC3988379

[B74] Nagano-Saito A. Cisek P. Perna A. S. Shirdel F. Z. Benkelfat C. Leyton M.. (2012). From anticipation to action, the role of dopamine in perceptual decision making: an fMRI-tyrosine depletion study. J. Neurophysiol. 108, 501–512. 10.1152/jn.00592.2011 22552189

[B75] Nassar M. R. Rumsey K. M. Wilson R. C. Parikh K. Heasly B. Gold J. I. (2012). Rational regulation of learning dynamics by pupil-linked arousal systems. Nat. Neurosci. 15, 1040–1046. 10.1038/nn.3130 22660479 PMC3386464

[B76] O'Reilly J. X. (2013). Making predictions in a changing world-inference, uncertainty, and learning. Front. Neurosci. 7:105. 10.3389/fnins.2013.00105 23785310 PMC3682109

[B77] Park I. M. Meister M. L. R. Huk A. C. Pillow J. W. (2014). Encoding and decoding in parietal cortex during sensorimotor decision-making. Nat. Neurosci. 17, 1395–1403. 10.1038/nn.3800 25174005 PMC4176983

[B78] Payzan-LeNestour E. Dunne S. Bossaerts P. O'Doherty J. P. (2013). The neural representation of unexpected uncertainty during value-based decision making. Neuron 79, 191–201. 10.1016/j.neuron.2013.04.037 23849203 PMC4885745

[B79] Ratcliff R. (1978). A theory of memory retrival. Psychol. Rev. 85, 59–108. 10.1037/0033-295X.85.2.59

[B80] Ratcliff R. McKoon G. (2008). The diffusion decision model: theory and data for two-choice decision tasks. Neural Comput. 20, 873–922. 10.1162/neco.2008.12-06-420 18085991 PMC2474742

[B81] Schall J. D. Purcell B. A. Heitz R. P. Logan G. D. Palmeri T. J. (2011). Neural mechanisms of saccade target selection: gated accumulator model of the visual-motor cascade. Eur. J. Neurosci. 33, 1991–2002. 10.1111/j.1460-9568.2011.07715.x 21645095 PMC3111938

[B82] Schroll H. Hamker F. H. (2013). Computational models of basal-ganglia pathway functions: focus on functional neuroanatomy. Front. Syst. Neurosci. 7:122. 10.3389/fnsys.2013.00122 24416002 PMC3874581

[B83] Shan Q. Ge M. Christie M. J. Balleine B. W. (2014). The acquisition of goal-directed actions generates opposing plasticity in direct and indirect pathways in dorsomedial striatum. J. Neurosci. 34, 9196–9201. 10.1523/JNEUROSCI.0313-14.2014 25009253 PMC6608360

[B84] Silberberg G. Bolam J. P. (2015). Local and afferent synaptic pathways in the striatal microcircuitry. Curr. Opin. Neurobiol. 33, 182–187. 10.1016/j.conb.2015.05.002 26051382

[B85] Simen P. (2012). Evidence accumulator or decision threshold-which cortical mechanism are we observing? Front. Psychol. 3:183. 10.3389/fpsyg.2012.00183 22737136 PMC3380269

[B86] Smith Y. Bevan M. Shink E. Bolam J. (1998). Microcircuitry of the direct and indirect pathways of the basal ganglia. Neuroscience 86, 353–387. 9881853 10.1016/s0306-4522(98)00004-9

[B87] Standage D. Blohm G. Dorris M. C. (2014). On the neural implementation of the speed-accuracy trade-off. Front. Neurosci. 8:236. 10.3389/fnins.2014.00236 25165430 PMC4131279

[B88] Sutton R. S. Barto A. G. (1998). Reinforcement Learning: An Introduction. Cambridge, MA: MIT press.

[B89] Tanaka M. (2007). Cognitive signals in the primate motor thalamus predict saccade timing. J. Neurosci. 27, 12109–12118. 10.1523/JNEUROSCI.1873-07.2007 17978052 PMC6673367

[B90] Tanaka M. Kunimatsu J. (2011). Contribution of the central thalamus to the generation of volitional saccades. Eur. J. Neurosci. 33, 2046–2057. 10.1111/j.1460-9568.2011.07699.x 21645100

[B91] Tumer E. C. Brainard M. S. (2007). Performance variability enables adaptive plasticity of “crystallized” adult birdsong. Nature 450, 1240–1244. 10.1038/nature06390 18097411

[B92] van Maanen L. Brown S. D. Eichele T. Wagenmakers E.-J. Ho T. Serences J.. (2011). Neural correlates of trial-to-trial fluctuations in response caution. J. Neurosci. 31, 17488–17495. 10.1523/JNEUROSCI.2924-11.2011 22131410 PMC6623798

[B93] Verbruggen F. Stevens T. Chambers C. D. (2014). Proactive and reactive stopping when distracted: an attentional account. J. Exp. Psychol. Hum. Percept. Perform. 40, 1295–1300. 10.1037/a0036542 24842070 PMC4120704

[B94] Verstynen T. D. (2014). The organization and dynamics of corticostriatal pathways link the medial orbitofrontal cortex to future behavioral responses. J. Neurophysiol. 112, 2457–2469. 10.1152/jn.00221.2014 25143543

[B95] Wall N. R. DeLaParra M. Callaway E. M. Kreitzer A. C. (2013). Differential innervation of direct- and indirect-pathway striatal projection neurons. Neuron 79, 347–360. 10.1016/j.neuron.2013.05.014 23810541 PMC3729794

[B96] Wei W. Rubin J. E. Wang X.-J. (2015). Role of the indirect pathway of the basal ganglia in perceptual decision making. J. Neurosci. 35, 4052–4064. 10.1523/JNEUROSCI.3611-14.2015 25740532 PMC4348195

[B97] Wiecki T. V. Frank M. J. (2013). A computational model of inhibitory control in frontal cortex and basal ganglia. Psychol. Rev. 120, 329–355. 10.1037/a0031542 23586447

[B98] Woolley S. C. Rajan R. Joshua M. Doupe A. J. (2014). Emergence of context-dependent variability across a basal ganglia network. Neuron 82 208–223. 10.1016/j.neuron.2014.01.039 24698276 PMC4132189

[B99] Wu H. G. Miyamoto Y. R. Gonzalez Castro L. N. Ölveczky B. P. Smith M. A. (2014). Temporal structure of motor variability is dynamically regulated and predicts motor learning ability. Nat. Neurosci. 17, 312–321. 10.1038/nn.3616 24413700 PMC4442489

[B100] Wu Y. Richard S. Parent A. (2000). The organization of the striatal output system: a single-cell juxtacellular labeling study in the rat. Neurosci. Res. 38, 49–62. 10.1016/S0168-0102(00)00140-1 10997578

[B101] Yanike M. Ferrera V. P. (2014). Interpretive monitoring in the caudate nucleus. Elife 3, 1–16. 10.7554/eLife.03727 25415238 PMC4238052

[B102] Yawata S. Yamaguchi T. Danjo T. Hikida T. Nakanishi S. (2012). Pathway-specific control of reward learning and its flexibility via selective dopamine receptors in the nucleus accumbens. Proc. Natil. Acad. Sci. U.S.A. 109, 12764–12769. 10.1073/pnas.1210797109 22802650 PMC3412032

[B103] Yoshida A. Tanaka M. (2016). Two types of neurons in the primate globus pallidus external segment play distinct roles in antisaccade generation. Cereb. Cortex 26, 1187–1199. 10.1093/cercor/bhu308 25577577

